# Preparation and characterization of HfOC/SiOC composite powders and fibermats *via* the polymer pyrolysis route†

**DOI:** 10.1039/d5ra02006a

**Published:** 2025-05-09

**Authors:** Arijit Roy, Paul Owiredu, Gurpreet Singh

**Affiliations:** a Mechanical and Nuclear Engineering Department, Kansas State University Manhattan KS 66506 USA arijit@ksu.edu gurpreet@ksu.edu

## Abstract

We report on the synthesis and characterization of HfOC/SiOC ceramic composite powders and electrospun fibermats, which integrate the high-temperature resilience of HfOC with the oxidation resistance of silicon oxycarbide (SiOC). The composites were fabricated through a polymer-pyrolysis route by integrating 1,3,5,7-tetramethyl, 1,3,5,7-tetravinyl cyclotetrasiloxane (4-TTCS), a precursor source for SiOC, and a commercial HfC precursor in a 1 : 1 ratio by mass. First, the HfC precursor was heated to 70 °C to drive off water molecules, followed by its blending with the liquid phase 4-TTCS and cross-linking at a moderate temperature (160–400 °C). This was followed by pyrolysis at three different temperatures – 800, 1000, and 1200 °C in an inert argon atmosphere. The composite ceramic was comprehensively characterized by the use of electron microscopy for particle and fiber morphology, X-ray diffraction for the evolution of various ceramic phases, and a range of spectroscopies to document the change in molecular vibrations or the evolution of the functional groups and molecular bonding in preceramic polymer during cross-linking and ceramization. The crosslinked polymer-to-ceramic yield for powder samples was observed to be as high as approximately 78 wt% when pyrolyzed at 800 °C, and 74 wt% when pyrolyzed at 1200 °C. The oxidation test performed at 800 °C in stagnant air for the fibermat pyrolyzed at 1000 °C indicated a linear shrinkage of 6% for the HfOC/SiOC composite. This represents an improvement over the carbon rich-SiOC fibermat which exhibited a mass loss of 71 wt% and a linear shrinkage of nearly 19%, while the neat carbon fibermat was completely burned off under similar conditions.

## Abstract 2

Synthesis and characterization of a HfOC/SiOC ceramic composite.

## Graphical abstract

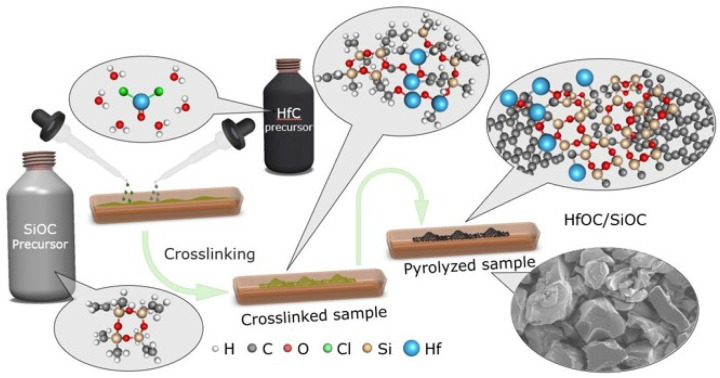

## Introduction

Ultra-high-temperature ceramics (UHTCs) possess remarkable durability in high-temperature applications, attributed to their elevated melting points, low coefficients of thermal expansion, and exceptional hardness.^1–3^ Typically, UHTCs consist of transition metal carbides, borides, and nitrides, such as hafnium carbide (HfC), zirconium diboride (ZrB_2_), and titanium nitride (TiN) among others.^4–6^ In particular, researchers have shown considerable interest in hafnium monocarbide, which exhibits one of the highest melting points at 3928 °C, as well as exceptional hardness (22–25 GPa) and Young's modulus (300–500 GPa).^7–9^ However, the low-fracture toughness (1.73–3.40 MPa √m) of HfC and its susceptibility to oxidation at high temperatures limits its applications.^10,11^ To address such issues, silicon carbide (SiC), silicon dioxide (SiO_2_), and silicon carbonitrides (SiCN) are typically incorporated into metal carbides to create composite ceramic matrices, which enhances mechanical properties compared to single-phase ceramic materials.^10,12–15^ This silicon-based framework improves intermolecular bonding interactions, thus increasing the oxidation resistance of the ceramic composite, rendering it suitable for aerospace applications.^16^

The ceramic composites are conventionally synthesized through solid-state sintering of metal carbide and silicon carbide powders, while sintering is challenging because the coarse particle size necessitates high temperatures, significant pressure, and extended production time.^17,18^ In contrast, a polymer-derived ceramic (PDC) offers an easier and more effective solution for dual-phase ceramic composites preparation with tailored phase composition and microstructures, leading to enhanced properties.^19,20^ Silicon oxycarbide (SiOC) is one of the earliest silicon-based PDCs, extensively studied for its available source of raw materials, cost-effectiveness, scalability in production, and significant design flexibility, which is conferred by the molecular structure of its precursors.^21–25^ Previous studies have demonstrated that the incorporation of SiOC with Hf to form a SiOC/HfO_2_ composite significantly enhances oxidation resistance at temperatures of up to 1600 °C, and this enhancement is attributed to the formation of HfSiO_4_ at elevated temperatures, which acts as a protective layer.^16,26^ This incorporation of hafnia (HfO_2_) within SiOC ceramic composites enhances thermal stability by inhibiting phase separation and decomposition while also improving creep resistance under stress at elevated temperatures compared to neat SiOC.^26–28^

Thus, a synergistic interaction between the protective properties of SiOC ceramics and the high-temperature stability of HfC ceramics could position HfOC/SiOC ceramics as ideal candidates for advanced high-temperature applications that require material stability in oxidative environments. In this study, liquid preceramic precursors of HfC and SiOC were employed to synthesize a HfOC/SiOC ceramic composite matrix. The pyrolyzed ceramic composite matrices at three different temperatures, in both particulate and fibrous forms, were analyzed to confirm the presence of HfC and SiOC ceramics, as well as HfO_2_ and excess carbon. The conversion of polymers to ceramics and the subsequent structural evolution of the customized nanoforms suggest that this method could be utilized for the development of a range of dual-phase ceramics, especially those with a silicon-based skeleton. Additionally, the oxidation analysis of the HfOC/SiOC fibers at 800 °C shows that incorporating Hf into the SiOC ceramic composite improves yields, while preserving the fiber structure.

## Material characterization

The morphology and microstructure of the HfOC/SiOC ceramics were characterized by utilizing a scanning electron microscope (SEM, SU8010, Hitachi). This microscope was also equipped with an X-ray fluorescence (XRF) analyzer for the determination of the elemental composition of the specimens. The presence and alteration in the chemical functional groups of polymer precursors and ceramic composite powder were assessed with Fourier-transform infrared spectroscopy (FTIR) conducted on a Thermo Scientific Nicolet iS5 (iD7 ATR) spectrometer. Surface chemical composition analysis was conducted *via* X-ray photoelectron spectroscopy (XPS) with a Thermo Scientific Al Kα^+^ source (beam energy = 1486.6 eV, spot size = 400 μm). A confocal Raman microscope (Renishaw inVia, wavelength 532 nm) was employed to determine the carbon vibrational modes in HfOC/SiOC ceramics. The crystallography of the material was analyzed through X-ray diffraction (XRD) utilizing a PANalytical Empyrean instrument set to 45 kV and 40 mA power, with a step size of 0.02°.

## Materials and methods

### Materials

The preceramic silicon oligomer 1,3,5,7-tetramethyl 1,3,5,7-tetravinyl cyclotetrasiloxane (denoted as 4TTCS) was purchased from Gelest (Morrisville, PA, USA). The crosslinking agent, [C_6_H_5_C(CH_3_)_2_]_2_O_2_ or bis(1-methyl-1-phenylethyl) peroxide or dicumyl peroxide (denoted as DCP), was purchased from Sigma-Aldrich (St. Louis, MO, USA).^29^ The spinning agent polyvinylpyrrolidone (PVP) with an average molecular mass of 1 300 000 g mol^−1^ was purchased from Sigma-Aldrich (St. Louis, MO, USA). The HfC precursor, SPH-199 HFC (Starfire Systems), was supplied by Spirit AeroSystems Inc. The ultra-high-purity argon (Ar) gas in the glove box and tube furnace was purchased from Matheson (Manhattan, KS, USA). The isopropyl alcohol or IPA was purchased from Thermo Fisher Scientific (Waltham, MA, USA).

### Methods

#### Preparation of ceramic particles

The synthesis of HfOC/SiOC ceramic particles was conducted in two stages. Initially, 5 mL of water-based HfC precursor was dried at 70 °C to get rid of excess water. Subsequently, it was added to 5 mL of the SiOC precursor, or 4TTCS, along with 1 wt% of DCP, and was subjected to magnetic stirring. Drying of HfC precursor was necessary because the HfC liquid precursor was found to be immiscible in liquid 4TTCS directly. The solution obtained was crosslinked at a temperature of 160 °C overnight on a hot plate inside a glove box filled with ultra-high-purity argon gas. The resulting brownish material is referred to as crosslinked or HfC/4TTCS XL particles. During this crosslinking process, in the presence of a crosslinking agent, the 4TTCS was immobilized, and a possible polymeric network of 4TTCS and HfC was formed.

**Fig. 1:**
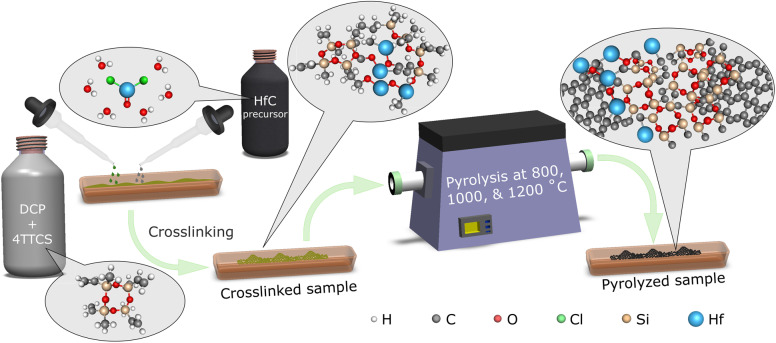
Schematic representation of the synthesis process for HfOC/SiOC ceramic particles. The HfC precursor and 4TTCS were first crosslinked and then pyrolyzed at three different temperatures to obtain HfOC/SiOC ceramic particles.

In the second stage, the crosslinked polymer was pyrolyzed (py) at three different temperatures (800, 1000, and 1200 °C) in a tube furnace with a continuous flow of Ar gas. The cross-linked samples were initially heated to 400 °C for 10 minutes at a rate of 2 °C per minute. Subsequently, the temperature was raised to 800 °C, 1000 °C, and 1200 °C at a rate of 5 °C per minute, and maintained for 1 hour to synthesize three different pyrolyzed samples. These obtained dark samples were denoted as HfOC/SiOC ceramic composite particles. The precision of the temperature controller in the tube furnace is ±1 °C. This high-temperature carbonization of the crosslinked polymers possibly transformed into HfOC/SiOC ceramic network along with excess carbon, originated from 4TTCS. The schematic in Fig. 1 demonstrates the synthesis process of the HfOC/SiOC ceramic particles.

#### Preparation of ceramic fibermats

The HfOC/SiOC ceramic fibermats were synthesized in three stages, beginning with the electrospinning stage. At first, 90 mg of crosslinked HfC/4TTCS was dispersed into 6 mL of IPA and sonicated (bath) for 30 minutes. Then 600 mg of PVP (with a PVP : IPA ratio of 100 : 1 mg mL^−1^) was added to the solution and magnetically stirred. After an hour, 1800 mg of 4TTCS (maintaining an XL-HfC/4TTCS : 4TTCS ratio of 1 : 20) and 18 mg of DCP (1 wt% of 4TTCS) were added, and the stirring continued overnight to confirm a homogeneous mixture. The resulting solution was loaded into a 10 mL syringe with a metallic needle for electrospinning. A 15 cm distance was kept between the needle tip of the syringe and the cylindrical-shaped roller collector, while maintaining a high applied voltage (∼15 kV) and slow feed rate (∼5 mL h^−1^). The as-spun fibermat (15 × 15 cm^2^) was dried in the open air at room temperature overnight and denoted as as-spun HfOC/SiOC FM. All mass measurements in this study were conducted utilizing a precision scale with a readability of 0.1 mg and a linearity of ±0.2 mg.

In the second stage, the fibermat was crosslinked at 160 °C for 6 hours in air, and in the final stage, the XL fibermats were cut into smaller pieces (∼5.5 × 3.5 cm^2^) and pyrolyzed at three different temperatures (800, 1000, and 1200 °C) in an inert atmosphere. This two-step annealing process started with increasing the temperature up to 400 °C at a heating rate of 2 °C min^−1^ and maintaining that for an hour. Then, similar to the particle samples, the temperature was raised to 800, 1000, and 1200 °C with a rate of 2 °C min^−1^, maintaining that for 1 hour for synthesizing three different HfOC/SiOC fibermats. The presence of PVP in this high-temperature treatment contributes to a higher quantity of free carbon in the fibermat samples compared to the particle form. All the crosslinking to pyrolysis yields for both the particle and fibermat samples are tabulated in Table S1.†

#### Sample preparation for oxidation of the fibermats

Along with HfOC/SiOC fibermats, PVP-derived carbonized and SiOC fibermats were prepared. These fibermats were prepared using similar conditions as the HfOC/SiOC fibermats, with the only difference in the solution for electrospinning. In the case of PVP-derived carbonized fibermat, there was no HfC or 4TTCS precursor (or XL material) present, and for SiOC fibermat, 4TTCS with DCP was added to the solution without any HfC precursor. The PVP-derived carbonized fibermat was synthesized *via* electrospinning on three occasions, yielding an average mass of about 300 mg (±15 mg), indicating minimal influence of repeated electrospinning processes on final characteristics of the fibermats under the same conditions. Then, circular-shaped fibermats with an area of 1.6 cm^2^ were punched out for the oxidation tests.

## Result and discussion

**Fig. 2:**
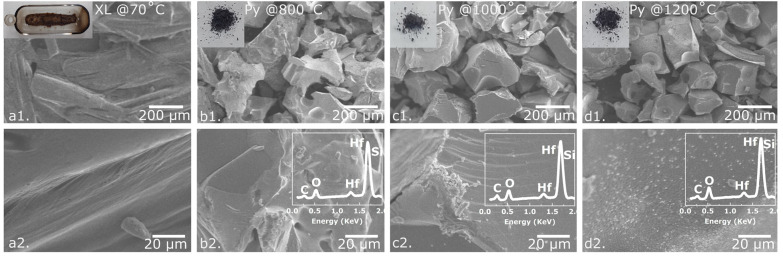
SEM images of (a1) low magnification crosslinked, (a2) high magnification crosslinked, (b1) low magnification pyrolyzed at 800 °C, (b2) high magnification pyrolyzed at 800 °C, (c1) low magnification pyrolyzed at 1000 °C, (c2) high magnification pyrolyzed at 1000 °C, (d1) low magnification pyrolyzed at 1200 °C, (d2) high magnification pyrolyzed at 1200 °C HfOC/SiOC ceramic particles and their corresponding camera images as inset of (a1–d1) and XRF data as inset of (b2–d2). The SEM images depict the particulate form and the change in average particle size of the HfOC/SiOC ceramic, while the XRF data confirms the presence of Hf and Si.

The SEM images of the crosslinked and pyrolyzed samples at various temperatures (800, 1000, and 1200 °C) have been shown in Fig. 2, and the insets of Fig. 2(a1–d1) display camera images of the corresponding samples. The SEM and camera pictures of crosslinked polymer samples consist of a compact morphology, whereas the pyrolyzed samples indicate uniform distribution of particles. The average particle size of the sample pyrolyzed at 800 °C was approximately 300 μm, and it decreased as the pyrolysis temperature increased. This morphological change supposedly indicates the radical conversion of HfO_2_ to HfC during pyrolysis.^30,31^Fig. 2(d2) shows that a high pyrolysis temperature of 1200 °C has resulted in increased porosity and a coarse surface for the ceramics. This is potentially caused by the release of volatile gases such as CO, CO_2_, and CH_4_.^31^ The XRF data of all the pyrolyzed HfOC/SiOC samples are presented in the inset of Fig. 2(b2–d2), which reveals the presence of Hf and Si (overlapped with Hf peak) with O and C. However, the XRF analysis has inherent limitations in detecting lighter elements accurately. In the presence of additional elements, the measured intensity of the target element may be influenced either through absorption or enhancement of its fluorescent X-rays, and this phenomenon is known as the matrix effect, which can result in inaccurate outcomes in XRF analysis. Hence, the XRF data utilized in this study were employed exclusively for the identification of material composition, rather than for quantification purposes.

**Fig. 3:**
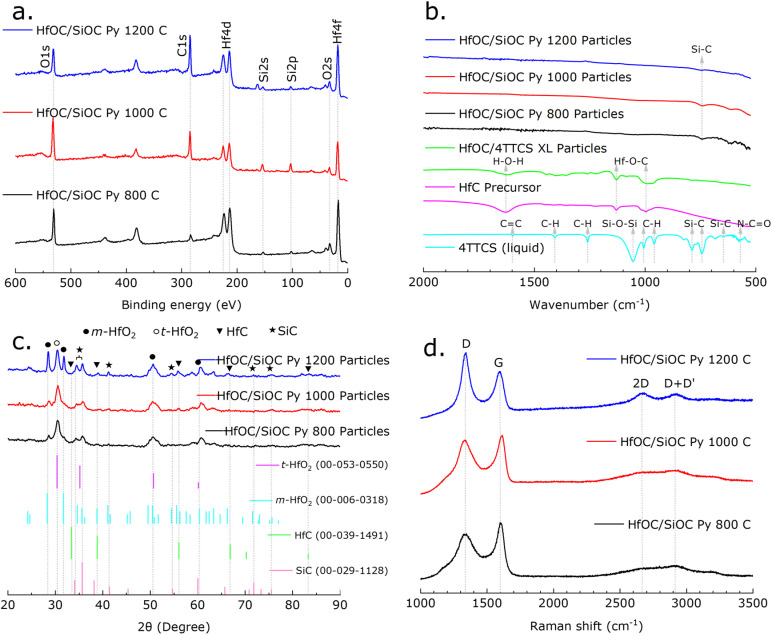
(a) The XPS survey scan of the HfOC/SiOC ceramic particles pyrolyzed at 800, 1000, and 1200 °C represents the binding energy and possible bonding between Hf, Si, O and C. (b) Their FTIR spectra, including precursors and crosslinked samples, represents the gradual disappearance of H-containing functional groups. (c) Their XRD pattern, including comparable JCPDS card numbers, indicates the increase in crystallinity with the increase in pyrolysis temperature. (d) The Raman spectra of HfOC/SiOC ceramic particles indicate evolution of the carbon phase as function of the pyrolysis temperature.

The chemical binding states of the HfOC/SiOC were analyzed using XPS survey scan, as shown in Fig. 3(a). The XPS data revealed that the composite sample contains Hf 4f, Si 2p, O 1s, and C 1s peaks. The Hf 4f peak at 18.08 eV binding energy suggests the presence of Hf–O–Si (hafnium silicate), Hf–C, and Hf–O bonds.^32,33^ The Si 2p peak at 103.08 eV reveals the Hf–O–Si, SiCO_3_, and SiC_2_O_2_ bonds.^10,34^ At 285.08 eV binding energy, the C 1s peak corresponds to C–O bond and C–C bond, confirming the presence of free carbon in the ceramic structure.^10^ The O 1s peak of the ceramic indicates the Si–O bond of SiO_*x*_ and C=O bonds at 532.08 eV.^32,35^ Also, the additional peaks for Hf 4d (214.08 and 225.08 eV) are associated with Hf–O bond of HfO_*x*_, and the presence of Si 2s (154.08 eV) and O 2s (33.08 eV) peaks suggests the formation of SiOC ceramic.^36,37^

The evolution of molecular structure and the presence of various functional groups were assessed through the analysis of FTIR spectra obtained from the 4TTCS and HfC precursors, along with their crosslinked and pyrolyzed particle samples, as illustrated in Fig. 3(b). For the 4TTCS or SiOC precursor, the peaks at approximately 1598, 1408, 1260, 1054, 1006, 958, 787, 743, 645, and 569 cm^−1^ correspond to the C=C stretching of Si–CH=CH_2_, C–H asymmetric bending of Si–CH_3_, C–H symmetric bending of Si–CH_3_, Si–O–Si asymmetric stretching, CH out-of-plane bending of Si–CH=CH_2_, CH out-of-plane bending of Si–CH=CH_2_, Si–C deformation vibration of Si–CH_3_, Si–C deformation vibration of Si–CH_3_, Si–C stretching of Si–CH_3_, and N–C=O bending, respectively.^34,35,38–40^ The FTIR spectra of the HfC liquid precursor show a broad H–O–H adsorption band near 1628 cm^−1^, indicating the bending vibrational mode of water molecules.^10^ The FTIR intensity indicates that following the crosslinking of HfC/4TTCS at 160 °C, there is a decrease in water molecule presence and an increase in Hf–O–C bonds, as evidenced by the intensified absorption peaks located near 1128 and 996 cm^−1^.^10,41^ Furthermore, minor peaks observed near 1400, 1260, and 780 cm^−1^ indicate C–H asymmetric bending, C–H symmetric bending, and Si–C deformation, respectively, confirming the presence of 4TTCS. After pyrolysis, only Si–C peaks were observed, while all characteristic bands associated with H-containing functional groups were absent, which suggests a complete transformation into ceramic material.^42^

The crystallographic characterization of the HfOC/SiOC ceramic samples was performed utilizing XRD, as presented in Fig. 3(c), revealing crystalline characteristics in the pyrolyzed samples. The XRD data of the synthesized ceramic samples were matched with reference pattern from ICDD database for tetragonal hafnia or t-HfO_2_ (JCPDS card no. 00-053-0550), monoclinic hafnia or m-HfO_2_ (JCPDS card no. 00-006-0318), HfC (JCPDS card no. 00-039-1491), SiC (JCPDS card no. 00-029-1128), Si (JCPDS card no. 01-089-2955), and C (JCPDS card no. 00-050-1082). Here, the diffraction peaks at 28.4°, 31.7°, 50.5°, and 60.3° on the 2*θ* scale correspond to (−111), (111), (−220), and (131) planes respectively of m-HfO_2_, while the peaks at 30.4° correspond to (111) plane of t-HfO_2_.^43^ The peak intensity of t-HfO_2_ remains unchanged as the pyrolysis temperature rises, while the intensity of m-HfO_2_ increases, indicating the conversion of t-HfO_2_ to m-HfO_2_.^16^ The (111), (200), (220), (311), and (400) planes of HfC were ascribed to the 33.4°, 38.8°, 56.0°, 66.8°, and 83.2°, respectively.^44^ The diffraction peaks at 34.2°, 35.6°, 41.4°, 54.6°, 71.7°, and 75.5° were assigned to the (101), (102), (104), (107), (202), and (204) crystalline planes of SiC, respectively.^45^ The intensity of SiC planes in the synthesized samples shows that higher pyrolysis temperatures lead to greater SiC crystallization.

The carbon vibrational modes of HfOC/SiOC ceramic particles were analyzed through Raman spectroscopy and illustrated in Fig. 3(d). The Raman spectra revealed two distinct peaks corresponding to the D (∼1320–1350 cm^−1^) and G (∼1589–1614 cm^−1^) bands. The D band was correlated with disordered carbon, which was characterized by lattice defects, displaying A_1g_ symmetry, and the G band was assigned to the in-plane stretching of sp^2^ hybridized carbon, exhibiting E_2g_ symmetry.^10,46^ In the ceramic particles pyrolyzed at 800 °C, the G band was observed to be more prominent than the broader D band, indicating a predominance of graphitic carbon in the sample. As the pyrolysis temperature rose, the D band became more distinct and prominent, while the G band expanded, signifying a rise in disordered carbon.^10^ In the second order of Raman spectra, the 2D peak (∼2660–2690 cm^−1^), identified as an overtone of the D peak, has been associated with the stacking of graphene layers in the structural composition, and the disordered graphitic sites correspond to the D + D′ band (∼2900–2930 cm^−1^).^47^

**Fig. 4:**
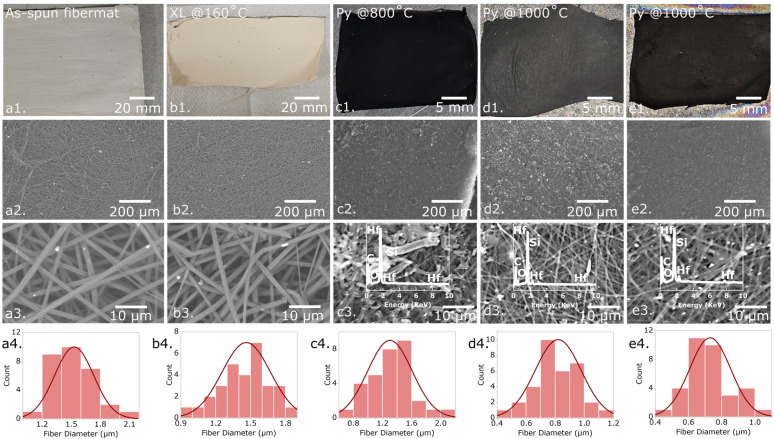
Camera pictures of (a1) as-spun, (b1) crosslinked, pyrolyzed at (c1) 800 °C, (d1) 1000 °C, and (e1) 1200 °C HfOC/SiOC ceramic fibermats; (a2) low and (a3) high magnification SEM images of as-spun, (b2) low and (b3) high magnification SEM images of crosslinked, (c2) low and (c3) high magnification SEM images of pyrolyzed at 800 °C, (d2) low and (d3) high magnification SEM images of pyrolyzed at 1000 °C, (e2) low and (e3) high magnification SEM images of pyrolyzed at 1200 °C HfOC/SiOC ceramic fibermats and their corresponding XRF data as inset of (c3–e3), representing the presence of Hf and Si in the fibers. The diameter distribution curves of (a4) as-spun, (b4) crosslinked, pyrolyzed at (c4) 800 °C, (d4) 1000 °C, and (e4) 1200 °C HfOC/SiOC ceramic fibermats illustrate a reduction in the average diameter of the fibers as pyrolysis temperature increases.

In this study, the HfOC/SiOC ceramic was examined both in particulate form and fibermat configuration. Fig. 4 presents the camera pictures, SEM images, and diameter distribution curves of the as-spun, crosslinked, and three distinct pyrolyzed HfOC/SiOC fibermat samples subjected to pyrolysis temperatures of 800, 1000, and 1200 °C. The average diameter of the as-spun fibermats was measured at approximately 1.55 μm, which subsequently reduced to ∼1.45 μm following crosslinking. Further reductions were observed, with diameters decreasing to approximately 1.3, 0.85, and 0.75 μm after pyrolysis at temperatures of 800, 1000, and 1200 °C, respectively. In addition, the XRF data for the three pyrolyzed fibermat samples are illustrated as the insets of Fig. 4(c3–e3), indicating a significant presence of Hf and Si (overlapped with Hf peak) similar to particle samples. However, in the fibermat form, carbon presence was significantly higher than in the particle form, despite having a similar intensity of the O peak, thereby confirming the presence of SiOC. The excess carbon in the pyrolyzed samples was primarily generated from the spinning agent of the fibermat or PVP.

**Fig. 5:**
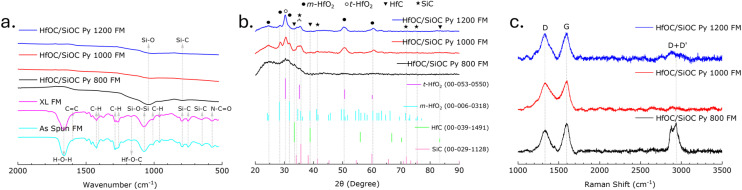
(a) The FTIR spectra of HfOC/SiOC ceramic fibermats pyrolyzed at 800, 1000, and 1200 °C, including as-spun and crosslinked fibermat samples, represent the gradual disappearance of H-containing functional groups. (b) Their XRD pattern, including comparable JCPDS card numbers, represents an increase in crystallinity with the increase of pyrolysis temperature. (c) Their Raman spectra indicate evolution of the free carbon phase as a function of pyrolysis temperature.

The FTIR spectra of as-spun, crosslinked, and pyrolyzed fibermat (at temperatures of 800, 1000, and 1200 °C) samples, are illustrated in Fig. 5(a). The chemical characteristics of the as-spun and crosslinked fibermat samples across various wavenumbers are detailed in Table 1. Notably, the intensity of the C–H bending reduced in the crosslinked samples in comparison to the as-spun samples, thereby confirming that the crosslinking reaction occurred at 160 °C.^34^ On the other hand, the pyrolyzed samples exhibited a more pronounced peak for the Si–O bond in comparison to the Si–C bond, thereby affirming the existence of SiOC ceramic within the sample. Also, the absence of all H-containing functional groups indicates that the material has entirely transformed into a ceramic state.^42^ However, as the pyrolysis temperature elevated from 800 °C to 1000 °C and 1200 °C, the observed peaks exhibited increased broadening and decreased intensity.

**Table 1:** FTIR spectra of the electrospun and crosslinked HfOC/SiOC fibermats

Wavenumber (cm^−1^)	Chemical group	Reference
1663	H–O–H (vibrational mode of water molecule)	10
1598	Si–CH=CH_2_ (C=C stretching)	34 and 39
1423	CH bending	34 and 38
1406	Si–CH_3_ (C–H asymmetric bending)	34 and 39
1286	CH_2_ wagging	34 and 38
1259	Si–CH_3_ (C–H symmetric bending)	34 and 39
1166	Hf–O–C	10 and 41
1070	Si–O–Si asymmetric stretching	34 and 39
1008	Si–CH=CH_2_ (CH out-of-plane bending)	34 and 39
960	Si–CH=CH_2_ (CH out-of-plane bending)	34 and 39
792	Si–CH_3_ (Si–C deformation vibration)	34 and 40
750	Si–CH_3_ (Si–C deformation vibration)	34 and 40
646	Si–CH_3_ (Si–C stretching)	34 and 39
571	N–C=O bending	34 and 38

The XRD analysis of pyrolyzed HfOC/SiOC ceramic samples indicates that crystallization peaks become sharp when the pyrolysis temperature exceeds 800 °C, as shown in Fig. 5(b). In contrast, the sample pyrolyzed at 800 °C exhibited broader low-intensity peaks in XRD, signifying its amorphous nature. This characteristic appeared in the fibermat form only and was absent in the particulate form of HfOC/SiOC ceramics. The XRD data were analyzed in comparison with the established reference database, as previously conducted with particle forms. The observed peaks at 24.6°, 28.4°, 31.7°, 50.5°, and 60.3° on the 2*θ* scale correspond to the (−110), (−111), (111), (−220), and (131) crystallographic planes of m-HfO_2_, respectively, and, the peak at 30.4° corresponds to the (111) plane of t-HfO_2_.^43^ In alignment with the XRD data pertaining to the particulate form, the peaks observed at 33.4°, 38.8°, and 83.2° correspond to the (111), (200), and (400) crystallographic planes of HfC, respectively.^44^ Additionally, the peaks identified at 34.2°, 35.6°, 41.4°, 54.6°, 71.7°, and 75.5° are associated with the (101), (102), (104), (202), and (204) crystallographic planes of SiC, respectively.^45^

The Raman spectra for the pyrolyzed fibermat samples are depicted in Fig. 5(c). These spectra exhibit peaks in the range of ∼1330–1340 cm^−1^ and ∼1589–1602 cm^−1^, corresponding to the D and G bands, respectively. Consistent with the particle form, the D band is associated with A_1g_ symmetry, indicating the presence of disordered carbon or out-of-plane vibrations, while the G band is linked to E_2g_ symmetry, which confirms the existence of C–C bonds associated with sp^2^ hybridization or in-plane stretching.^10,46^ In the fibermat samples also, the intensity of the D band increased with higher pyrolysis temperatures, suggesting an increase in disordered carbon.^10^ However, in the second-order Raman spectra, the 2D peak was minimized in all samples, while a distinct peak at ∼2938 cm^−1^ representing the D + D′ band was observed exclusively in the fibermat sample pyrolyzed at 800 °C.^47^

**Fig. 6:**
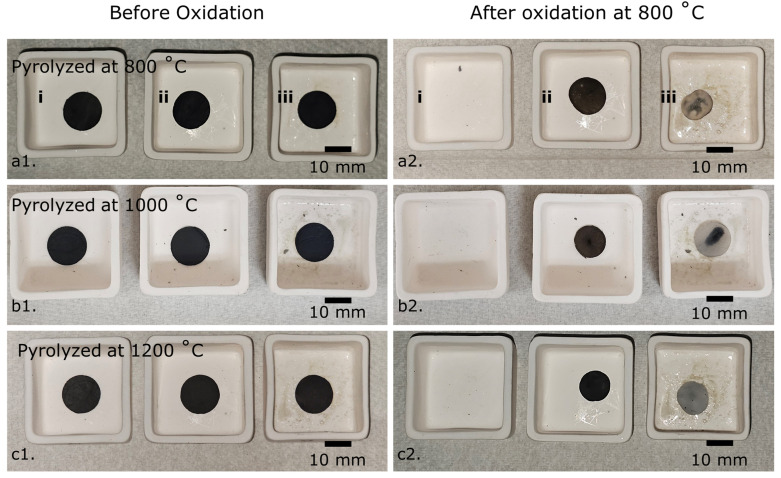
Camera images of various fibermat samples (a1–c1) before and (a2–c2) after the oxidation tests: (i) PVP-derived carbonized fibermats, (ii) carbon rich SiOC fibermats, and (iii) HfOC/SiOC fibermats. A summary of linear shrinkage for these samples is presented in the ESI.†

**Fig. 7:**
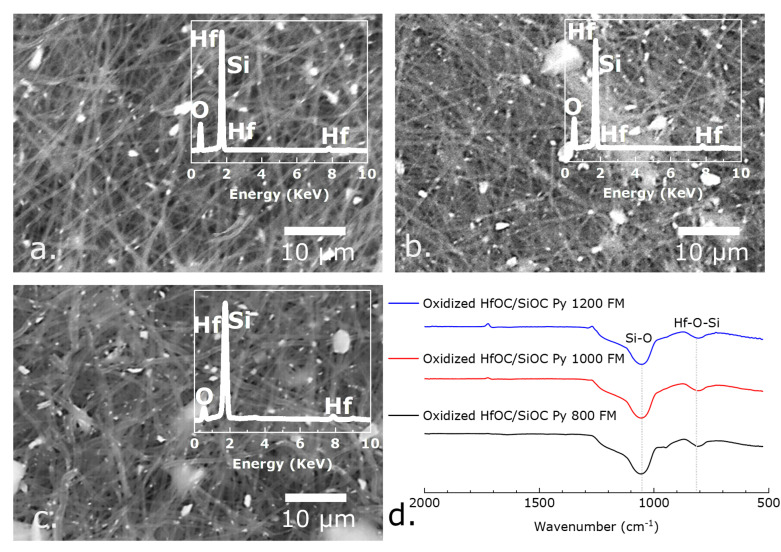
The SEM images of oxidized HfOC/SiOC ceramic fibermats at 800 °C for the samples pyrolyzed at (a) 800 °C, (b) 1000 °C, and (c) 1200 °C represent the fiber structure was preserved, while their corresponding XRF data, inset of (a–c), indicates the presence of Hf and Si. (d) The FTIR spectra of oxidized fibermats, pyrolyzed at 800, 1000, and 1200 °C, reveal the presence of vibrational modes for Si–O and Hf–O–Si.

Oxidation resistance behavior of the HfOC/SiOC ceramic fibermats along with PVP-derived carbonized and SiOC fibermats, for comparative analysis, were investigated by heating the circular-shaped samples (pyrolyzed fibermat at 800, 1000, and 1200 °C) in stagnant air at 800 °C for a duration of 5 minutes, employing a heating rate of 10 °C min^−1^. The camera pictures of the oxidized fibermats are presented in Fig. 6(a1–c2), with the SEM images of the oxidized HfOC/SiOC samples displayed in Fig. 7(a–c). The results indicate that, despite undergoing oxidation at 800 °C, the fiber structures remain intact in the cases of HfOC/SiOC and SiOC fibermats, whereas the PVP-derived carbonized fibermat did not survive. The carbonized samples produced little or no residue following the oxidation test, which aligns with the TGA analysis of previously reported studies.^48,49^ The mass losses due to oxidation of the fibermat samples are presented in Table S2.† The enhanced oxidation yield observed in the HfOC/SiOC fibermat (pyrolyzed at temperatures exceeding 800 °C) compared to that of the bare SiOC fibermat under similar conditions signifies a superior endurance attributed to the incorporation of Hf. Additionally, the linear shrinkage analysis (Fig. S1†) from the camera images (Fig. 6) shows that after the oxidation test, although the HfOC/SiOC fibermats show a slight tendency to curl, the shrinkage of the disk diameter is significantly lower than that of the SiOC fibermat when subjected to pyrolysis at temperatures above 800 °C. The reduction in thickness observed in the pre- and post-oxidation tests of the HfOC/SiOC fibermat samples is presented in Table S3.† The data reveal that when the fibermat sample undergoes pyrolysis at higher temperatures, such as 1200 °C, the reduction in thickness is limited to around 20%, in contrast to the ∼67% reduction observed when the pyrolysis temperature is 800 °C. The XRF data for the oxidized HfOC/SiOC ceramic fibermat samples were also analyzed and are presented in the inset of Fig. 7(a–c). As anticipated, the XRF data reveal no C peaks and a more pronounced O peak following oxidation across all samples. Nonetheless, the detection of Hf, Si, and O peaks demonstrates the endurance of the ceramic fiber forms for high temperature applications.

FTIR analysis was also used to identify the chemical characteristics of the oxidized fibermat samples, as illustrated in Fig. 7(d). In the wavenumber scale, two prominent peaks at approximately 1040–1060 cm^−1^ and 810–820 cm^−1^ signify the vibrational modes of Si–O and Hf–O–Si in the oxidized samples, respectively.^34,50^ This indicates that in the oxidation test, the Hf–C and Si–C bonds were disrupted, resulting in the formation of Hf–O and Hf–O–Si bonds while preserving the fiber structure.

The studies previously reported regarding Hf-induced polysilazane-based PDCs demonstrate a crosslink-to-pyrolysis mass loss of approximately 20% for particulate samples, which is consistent with the findings of the current research.^51^ In case of fibermat samples, the observed post-pyrolysis reduction of approximately 30–40% in the average fiber diameter further corroborates the data from earlier studies.^52^ The mass loss during oxidation tests typically ranges from approximately 20% to 57% for similar electrospun PDC nanofibers.^53^ Additionally, previous studies have demonstrated that Si/O/C type ceramics exhibit relatively low fracture toughness, such as PDC SiOC (0.88–0.99 MPa √m), SiO_2_ (0.52–1.02 MPa √m), SiC (1.4–1.9 MPa √m), compared to HfC incorporated silicon-based ceramic composite matrix (0.8–3 MPa √m); which would be the expected range of fracture toughness for the HfOC/SiOC samples of this study.^54–58^ Also, low thermal expansivity (3.5 × 10^−6^ K^−1^), moderate stiffness (1–8 GPa), and high strength (1.5–13 MPa) of SiOC offer better thermal shock resistance and fatigue resistance compared to similar PDCs.^59,60^ Previous studies indicate that the incorporation of HfC into silicon-based ceramics has been shown to effectively diminish the ablation rate of the composite material.^61–64^ Consequently, this study anticipates enhanced overall thermophysical properties of the HfOC/SiOC ceramic composite.

## Conclusion

This study presents a simple methodology for synthesizing HfOC/SiOC ceramic composites, in both particulate and fiber forms, by blending and pyrolysis of liquid 4TTCS and HfC precursors. The ceramics were synthesized from crosslinked polymers at three different temperatures: 800, 1000, and 1200 °C. The SEM images and XRF data demonstrate that the produced ceramic samples contain elements of Hf, Si, O, and C, confirming the successful formation of HfOC/SiOC ceramics, along with the excess or the free carbon phase in both particle and fiber configurations. The XPS analysis further validated these findings, while FTIR confirmed the organic to inorganic transformation of the polymer precursor into ceramic matrices. Further, the XRD analysis confirmed the evolution of HfC, monoclinic-HfO_2_, and SiC crystalline phases in specimen processed beyond 800 °C. Although the particle form exhibited X-ray crystallinity when subjected to pyrolysis at 800 °C, the fiber form remained largely X-ray amorphous and only started to crystallize at 1000 °C. Furthermore, oxidation tests conducted on the fiber form samples indicate that HfOC/SiOC ceramic fibermats exhibit improved thermal endurance for high-temperature applications. Post-oxidation analysis at 800 °C showed the retention of fiber forms, demonstrating superior performance (mass retention and shrinkage) due to the presence of Hf in the composite ceramic fibermats compared to bare PVP-derived carbonized and/or TTCS derived SiOC ceramic fibermats. This study does not concentrate on other mechanical and thermophysical properties; however, future research may encompass such investigations on this material. Furthermore, by varying the ratio of HfC to TTCS precursors, subsequent studies could focus on identifying the optimal material characteristics for specific high-temperature applications. This synthesis approach for the preparation of hybrid ceramics from the blending of precursors may have applications in the processing of other multi-elemental and high-performance ceramic composites.

## Data availability

Data for this article, including plots, are available at the Ondrive of Kansas State University at [URL – https://ksuemailprod-my.sharepoint.com/:f:/g/personal/arijit_ksu_edu/Ejy5zVytaQpIii8P0rAdcIgBQcNCMgLfgugeClcRVf_lpg?e=4fMPye].

## Author contributions

Conceptualization: G. S.; methodology: G. S., P. O., and A. R.; HfOC/SiOC particle synthesis, SEM, XRF, XPS, FTIR, XRD, and Raman: A. R. and P. O.; HfOC/SiOC fibermat synthesis, oxidation test, SEM, XRF, FTIR, XRD, and Raman analysis: A. R.; resources: G. S.; data curation: P. O. and A. R.; writing – original draft preparation: A. R. and P. O.; writing – review and editing: G. S.; visualization: A. R.; supervision: G. S.; funding acquisition: G. S. All authors have read and agreed to the published version of the manuscript.

## Conflicts of interest

There are no conflicts to declare.

## Supplementary Material

RA-015-D5RA02006A-s001
