# Supplementary material for: Preparation and characterization of HfOC/SiOC composite powders and fibermats via the polymer pyrolysis route
Source: RSC Adv. 2025 May 9;15(19):15397–407. doi: 10.1039/d5ra02006a (PMC12063723; doi:10.1039/d5ra02006a)
Supplement: RA-015-D5RA02006A-s001 [file RA-015-D5RA02006A-s001.pdf]

Supplementary Information (SI) for RSC Advances.
This journal is © The Royal Society of Chemistry 2025

Supporting information

Preparation and characterization of HfOC/SiOC Composite Powders and Fibermats via the Polymer Pyrolysis Route

Arijit Roy[1†], Paul Owiredu[1†], Gurpreet Singh[1*]

[1]Mechanical and Nuclear Engineering Department, Kansas State University, Manhattan, KS 66506, USA

[†]Equal contribution

Email: arijit@ksu.edu (A Roy), gurpreet@ksu.edu (G Singh)

Table S1: Crosslink to pyrolysis yield of HfOC/SiOC particles and fibermats pyrolyzed at different temperatures.

Samples	Yield at 800 ˚C	Yield at 1000 ˚C	Yield at 1200 ˚C
HfOC/SiOC particles (wt. %)	77.6	77.6	74.2
HfOC/SiOC fibermat (wt. %)	22.7	20.3	18.5
HfOC/SiOC fibermat (area %)	29.2	29.3	26.5

Table S2: Oxidation mass loss of ceramic fibermat samples.

Sample Name	Mass loss for py at 800 ˚C (wt.%)	Mass loss for py at 1000 ˚C (wt.%)	Mass loss for py at 1200 ˚C (wt.%)
SiOC	41	70.6	67.5
HfOC/SiOC	53.5	58.3	55.3

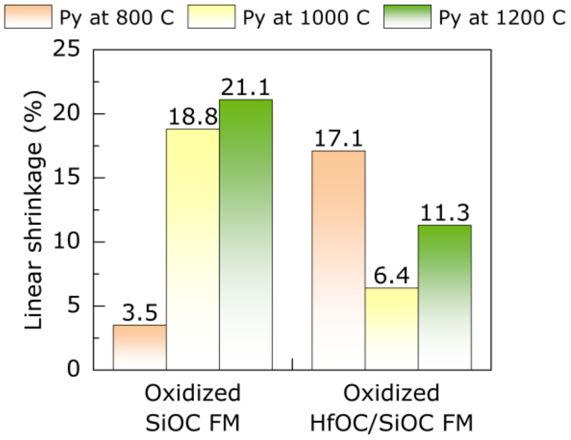

Figure S1: Linear shrinkage (in diameter) analysis for 800 ˚C oxidization of PVP, SiOC, and HfOC/SiOC fibermats.

Table S3: Thickness of the pre- and post-oxidized HfOC/SiOC fibermats.

HfOC/SiOC sample pyrolyzed at	Pre-oxidization test thickness (μm)	Post-oxidization test thickness (μm)	Reduction in thickness (%)
800 ˚C	220	70	68
1000 ˚C	140	70	50
1200 ˚C	100	80	20
